# False Passage to the Trachea after Emergency Intubation in a Victim of Near Hanging

**DOI:** 10.1155/2013/281307

**Published:** 2013-05-13

**Authors:** Ali Pourmand, Hamid Shokoohi

**Affiliations:** Department of Emergency Medicine, George Washington University Medical Center, 2120 L Street NW, Suite 450, Washington, DC 20037, USA

## Abstract

Emergency medicine physicians should have enough knowledge and experience to deal with emergent and traumatic difficult airway. In this paper, we present a case of near hanging with neck soft tissue injury, tracheal and esophageal rupture that is complicated by a displaced intubation and false passage to the trachea.

## 1. Introduction

Recent studies showed that hanging has become the third most cause of suicide attempts among young adults [1]. Victims of near hanging have a wide variety of clinical manifestations and complications [2]. Emergency physicians and trauma surgeons commonly encounter theses complications. We describe a case of near hanging with neck soft tissue injury that is complicated by a displaced intubation and false passage to the trachea.

## 2. Case Report

**Figure 1:**
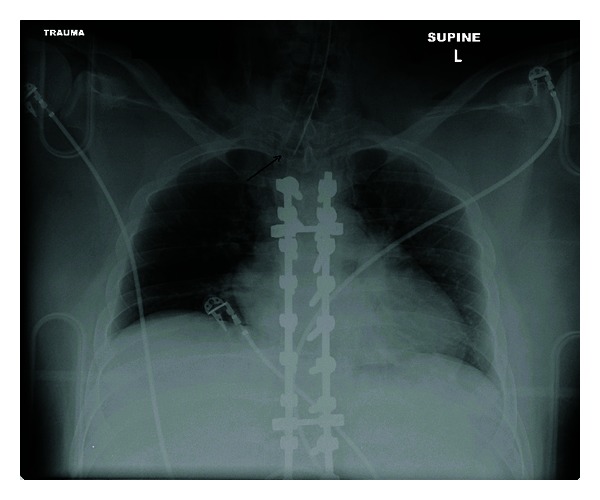
Chest X-ray after reintubation. Arrow: tip of ETT is at the thoracic inlet.

**Figure 2:**
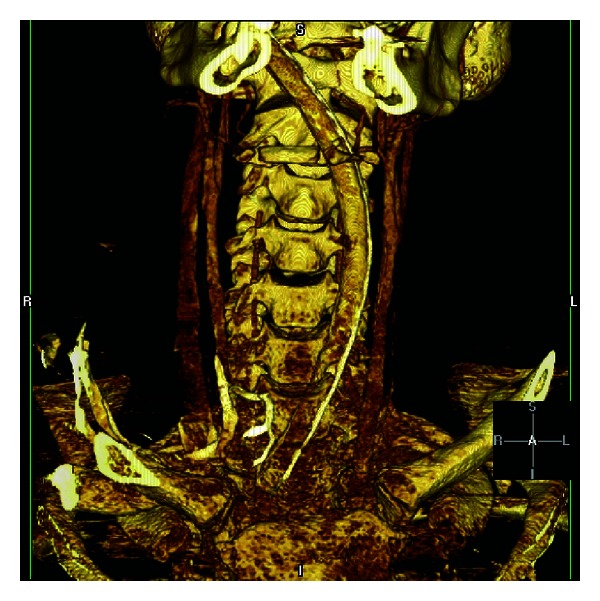
Three-dimensional CT scan of neck.

A 20-year-old Caucasian man was brought to the emergency department (ED) after having a suicidal attempt. He had a long history of depression and obsessive/compulsive disorder and came to Virginia with the stated intent to buy a gun because the gun laws were lenient in Virginia, and the intent of buying the gun was to kill himself. However, he did not buy a gun but did attempt suicide by cutting his wrists, then tying a rope around his neck, tying the other end of the rope to a tree, getting in his car, and driving away. The patient then got out of the car, walked to a nearby building, complained that he could not breathe, and then subsequently collapsed. When EMT arrived, he was intubated at the scene and then transferred to the hospital by air care. Upon arrival and evaluation in the ED, he was noted to have massive subcutaneous emphysema in the neck (Figure 1). The anesthesiologist present at the bedside felt that the endotracheal tube was not in the correct position, therefore, he decided to change the endotracheal tube with a new tube. The patient went under portable chest X-ray,and the radiologist mentioned that tip of tracheal tube is at thoracic inlet (Figure 1). The patient then went to radiology and had a CT scan of the head, neck, and chest which showed that the endotracheal tube is entering through the oropharynx, but in the hypopharynx, the tube appears to enter into the left piriform sinus, at which it would appear to leave the airway in the soft tissues of the neck on the left side and to reenter the trachea at the thoracic inlet. As visualized on the coronal images, the endotracheal tube had bypassed the area of vocal cords and upper trachea. This implies a disruption of the airway. There is diffuse subcutaneous air within the neck dissecting bilaterally and anteriorly as well. The endotracheal tube cuff is inflated. This may lie within the airway at approximately the level of the thyroid gland (Figure 2). The patient underwent bronchoscopy which did confirm that endotracheal tube eventually reentered the trachea distal to where it was coarse outside the trachea. Later he underwent an esophagography performed under fluoroscopy guidance, followed by an EGD in the operating room, which showed an additional rupture of the esophagus.

During his hospitalization, the patient underwent a crico-tracheal reanastomosis, open reduction and internal fixation of the cricoid fracture, direct esophageal repair and tracheostomy placement. An MRI of the cervical spine detected a prevertebral soft tissue swelling at the level of C2-C3 which could potentially represent a ligamentous injury to the C-spine but could also represent soft tissue swelling related to the nature of the injury. 

This patient was admitted to the ICU, where his course was complicated by sepsis and multiple infections. The patient was transferred to the intermediate critical care (IMC) and then psychiatric unit where they transferred patient to a partial day program in New York on the hospital day 74. He had an appointment with a psychiatrist and with a pulmonologist after leaving the hospital.

## 3. Discussion 

Major complications and injuries to the airway following hanging are common [2]. Complications related specifically to airway include air leaks, tracheal perforation, fractures of the hyoid bone, and cricoid cartilage and laryngeal injuries [3, 4].

Further iatrogenic airway injury from the insertion of an endotracheal tube during intubation may cause a significant morbidity in these cases with hanging injuries [5–7]. Creating a false passage in the cervical soft tissue during intubation can occur, but creating a passage that returns to the trachea is quite rare and often goes undiagnosed. Air leaks at the site of tracheal perforation and ETT malpositioning in the imaging studies are clues to detect this complication.

Obtaining a definitive airway in the victims of hanging with a compromised airway and neck injuries could be extremely difficult.

In our case, endotracheal intubation in the field was preferred because the patient was unconscious with an unstable hemodynamic status and obvious risk of losing airway as a result of further edema and an increased pressure to the airway. A good result was achieved with this out-of-hospital management as patient was successfully ventilated, but the sequential iatrogenic injury was created.

In this case, the patient could be easily ventilated because the endotracheal tube tip was returned to the trachea at the thoracic inlet after passing through the false passage. Massive subcutaneous emphysema and CT scan finding were guided toward the possibility of tracheal rupture that later was confirmed and repaired in bronchoscopic intervention.

Tracheal injury related to emergency intubations as the results of tube-cuff overinflation, direct puncture by the use of a stylet, and multiple attempts at intubation has been reported in different studies [8–10]. However the victims of hanging with preexisting neck injuries carried a higher risk for these iatrogenic complications.

In conclusion, it is considered that this complication, which has not been reported in the literature, should be kept in mind during the placement of ETT, and it should also be remembered that airway management with noninvasive methods might be equally successful.
